# Phenotypic and functional disparities in perivascular adipose tissue

**DOI:** 10.3389/fphys.2024.1499340

**Published:** 2024-11-11

**Authors:** Erling Guo, Dan Liu, Ziming Zhu

**Affiliations:** ^1^ College of Physical Education, Guangzhou College of Commerce, Guangzhou, China; ^2^ Primary Department International Division, Tsinghua International School Daoxiang Lake, Beijing, China; ^3^ International College, Guangzhou College of Commerce, Guangzhou, China

**Keywords:** perivascular adipose tissue, nitric oxide, adiponectin, β3-adrenoceptors, norepinephrine

## Abstract

The adipose tissue surrounding blood vessels is known as perivascular adipose tissue (PVAT), which represents a distinct ectopic fat depot that adheres to the majority of the vasculature. In recent years, owing to its unique location and function, PVAT has been regarded as a new type of adipose tissue distinct from traditional visceral fat. It releases adipokines with vasoconstrictive functions, which regulate vascular function through paracrine and endocrine mechanisms. Interestingly, PVAT can be categorized as white, brown or a mixture of both depending on its anatomical location. Brown adipose tissue (BAT) is located adjacent to the thoracic aorta in rodents, while a mix of brown and white tissue surrounds the abdominal aorta. PVAT exhibits regional phenotypic differences in different parts of the vasculature bed, which may lead to heterogeneity in the secretion profiles and norepinephrine (NE) content in regional PVAT and subsequently affect the regulation of specific adipokine signaling pathways in regional PVAT, resulting in differences in the regulation of vascular function. The aim of this review was to explore the potential factors that influence the anticontractile function of regional PVAT in the vasculature, including the heterogeneity of regional PVAT, the anticontractile function mediated by endothelial nitric oxide synthase (eNOS) in regional PVAT, the activity of the adiponectin-eNOS pathway in regional PVAT adipocytes, and the concentration of the sympathetic neurotransmitter NE in regional PVAT.

## 1 Introduction

The adipose tissue surrounding blood vessels is known as perivascular adipose tissue (PVAT), a distinct ectopic fat depot that adheres to the majority of the vasculature. Initially, PVAT was believed to serve only as a structural support tissue that provides physical protection to blood vessels (Antoniades et al., 2023). However, owing to its close proximity to the vasculature system and direct contact with the adventitia (Eringa et al., 2007), PVAT has increasingly been shown to play an important role in maintaining vascular homeostasis (Koenen et al., 2021). The involvement of PVAT in vascular function was initially identified by the observation that PVAT reduced the contractile response to noradrenaline (NA) in the aortas of rats (Soltis and Cassis, 1991). As a highly metabolically active tissue, PVAT regulates vascular tone by releasing numerous vasoactive factors (Rajsheker et al., 2010; Van de Voorde et al., 2013). Interestingly, regional variations in the phenotypic and functional characteristics of PVAT exist throughout different segments of the vascular system vasculature. The PVAT in the thoracic aorta exhibits characteristics of brown adipose tissue (BAT), which produces heat and exerts anti-inflammatory and anti-atherosclerotic effects (Police et al., 2009; Fitzgibbons et al., 2011; Padilla et al., 2013; Qi et al., 2018). The PVAT in the abdominal aorta shows characteristics of beige adipose tissue, which is composed of white adipose cells and a few brown adipose cells (Padilla et al., 2013; Li et al., 2021), and stores energy, but has a storage capacity that is lower than that of white adipose tissue (WAT) (Min et al., 2016). The PVAT in different regions significantly differs not only in terms of adipose tissue phenotype and function but also in terms of the degree of immune cell infiltration, response to different agonists, ability to release specific adipokines, and nerve innervation. The high heterogeneity of PVAT in the thoracic and abdominal regions may lead to differences in the anticontractile function of PVAT. In this review, we explore the potential factors that may contribute to the anticontractile function of regional PVAT from four aspects: heterogeneity in regional PVAT, the endothelial nitric oxide synthase (eNOS)-mediated anticontractile function of regional PVAT, adiponectin-eNOS pathway activity in regional PVAT, and the norepinephrine (NE) concentration in regional PVAT.

### 1.1 Heterogeneity of regional PVAT

Adipose tissue (AT) is a dynamic organ distributed throughout the body (Koenen et al., 2021). Depending on their anatomical location, these tissues exhibit distinct morphologies and functions. For example, visceral AT is a secretory AT reservoir that promotes atherosclerosis, whereas subcutaneous AT is a classic energy storage depot (Antoniades et al., 2023). PVAT was initially believed to primarily serve as a structural support tissue that provides physical protection to blood vessels (Antoniades et al., 2023). However, owing to its close proximity to the vasculature system and direct contact with the adventitia (Eringa et al., 2007), PVAT has increasingly been shown to play an important role in maintaining vascular homeostasis (Koenen et al., 2021). Interestingly, the vascular bed PVAT in the thoracic and abdominal regions show regional phenotypic differences. The PVAT surrounding the thoracic aorta exhibits phenotypic characteristics of BAT, and that surrounding the abdomen is a mix of brown and white tissue (Gao, 2007; Brown et al., 2014). Brown adipocytes are distinguished by multilocular lipid droplets and a high density of mitochondria, which uniquely express uncoupling protein-1 (UCP1) in the inner mitochondrial membrane. The oxidative respiratory chain from the electron transport chain within mitochondria is uncoupled to initiate thermogenesis (Qi et al., 2018). In contrast, beige adipocytes are characterized by the presence of large unilocular lipid droplets, lower mitochondrial numbers, and relatively small cytoplasmic volumes (Padilla et al., 2013; Brown et al., 2014; Li et al., 2021), resulting in a lower energy storage capacity than WAT and a lower heat production capacity than BAT (Min et al., 2016). In addition to functional differences, the PVAT of the thoracic and abdominal regions substantially varies in terms of the amount and size of adipocytes. As an extension of the thoracic aorta, the abdominal aorta is surrounded by 4–10 times more AT than the aortic arch in rats (Henrichot et al., 2005). Compared with that in thoracic PVAT, the size of adipocytes in abdominal PVAT was significantly greater. Importantly, these differences were independent of age-related changes in gene expression (Padilla et al., 2013). These findings further support the morphological, histological and functional differences in the PVAT between the thoracic and abdominal regions. Moreover, the thoracic PVAT and abdominal PVAT differ in the extent of immune cell infiltration. In addition to adipocytes, PVAT includes various cell types, including immune cells (Kumar et al., 2020). Under healthy conditions, macrophages and T cells constitute the main immune cell types in PVAT, which, together with eosinophils, are involved in the anti-inflammatory and anticontractile effects of PVAT on the vascular wall (Gil-Ortega et al., 2015; Withers et al., 2017). Studies have shown that the levels of inflammatory genes and immune cell infiltration markers are greater in abdominal PVAT than in thoracic PVAT (Police et al., 2009). The abdominal PVAT phenotype is more proinflammatory and proatherosclerotic than the thoracic PVAT phenotype is, which may explain the increased susceptibility to diseases in the abdominal aorta region (Police et al., 2009; Fitzgibbons et al., 2011; Padilla et al., 2013).

Interestingly, in obese rodents, the regional heterogeneity of PVAT becomes even more pronounced. According to observations in obese mice, excessive triacylglycerol storage in abdominal and thoracic fat leads to an increased prevalence of white adipose phenotypes (Fitzgibbons et al., 2011; Fernández-Alfonso et al., 2013; Gozal et al., 2017), accompanied by an increase in the mass and adipocyte size of PVAT (Marchesi et al., 2009; Ketonen et al., 2010). The adipocytes in the thoracic PVAT undergo transformation toward a “whiter” phenotype that is predominantly characterized by a unilocular morphology and enlarged lipid droplets (Gálvez-Prieto et al., 2008; Sacks and Symonds, 2013). In contrast, in abdominal PVAT, the susceptibility to lipid accumulation increases, and adipocytes exhibit distinct characteristics of hyperplasia and hypertrophy (Henrichot et al., 2005). In addition, the several proinflammatory chemokines and macrophage markers are significantly upregulated in thoracic PVAT and abdominal PVAT (Police et al., 2009). However, the inflammatory response in thoracic PVAT is substantially lower than that in abdominal PVAT (Police et al., 2009; Padilla et al., 2013). Moreover Li et al. (2021). revealed that after 4 months of high-fat diet feeding, the expression of inflammatory factor genes was greater in abdominal PVAT than in thoracic PVAT. The differences in the responses of AT in the thoracic and abdominal regions to obesity suggest that thoracic PVAT is more resistant to diet-induced inflammation than abdominal PVAT is. Together, these data suggest that the morphological, histological and functional features and differences in immune cell responses to PVAT in the thoracic and abdominal regions may be related to differences in their anticontractile ability.

### 1.2 The anticontractile function of regional PVAT

The involvement of PVAT in vascular function was initially identified by the observation that PVAT reduced the contractile response to NA in the aortas of rats (Soltis and Cassis, 1991). However, notably, the heterogeneous characteristics of PVAT in different regions of the aorta give rise to regional variations in the regulation of vascular function. The results of a study evaluating the effects of a high-fat diet on endothelial responses in the thoracic and abdominal aorta suggest that PVAT exhibits distinct endothelial responses in the thoracic and abdominal regions. Impaired acetylcholine (Ach)-induced vasodilation in the endothelium of the abdominal aorta was observed after only 2 weeks of high-fat diet feeding; this endothelial dysfunction subsequently progressed to the development of arterial stiffness, and an increased pulse wave velocity (PWV) was observed after 4–8 weeks of high-fat diet feeding. The aforementioned pathological changes in the endothelium, however, were not observed in the thoracic aorta, indicating that distinct PVAT depots exerted opposing effects on endothelial function in the aorta under high-fat diet conditions (Bar et al., 2020), thereby affecting nitric oxide (NO)- mediated endothelium-dependent vasodilation.

In addition, thoracic PVAT and abdominal PVAT differ in vascular tone responses induced by several systolic agonists. A study conducted by Victorio et al. (2016). revealed that PVAT reduces phenylephrine-induced contraction in the thoracic aorta, both in the absence and presence of endothelial cells. However, this anticontractile effect was not observed in the abdominal aorta. Additionally, the response to angiotensin II-induced contraction revealed that thoracic PVAT attenuated angiotensin II-induced contraction, whereas no attenuation was observed in abdominal PVAT (Watts et al., 2011). These findings further validate the significant difference in the anticontractile function of PVAT between the thoracic and abdominal regions. Victorio et al. (2016) analyzed the mechanisms underlying local functional differences by comparing the synthesis and availability of NO. The study findings suggest that the generation of reactive oxygen species (ROS) and lipid peroxidation in the abdominal and thoracic aorta and PVAT are similar, while endothelial nitric oxide synthase (eNOS) and NO availability in abdominal PVAT is lower than that in thoracic PVAT, but no changes were observed in the vasculature (Victorio et al., 2016). Therefore, the regional variability in eNOS-derived NO production by PVAT adipocytes may contribute to differences in their anticontractile function. However, a previous study showned that inhibiting NOS does not block the anticontractile function of aortic PVAT. Consequently, researchers concluded that the relaxation factor derived from PVAT is not NO (Löhn et al., 2002). As a highly active endocrine organ, PVAT actively releases numerous vasoactive factor molecules to regulate vascular function. However, depending on the vascular region, PVAT may have a heterogeneous ability to synthesize and secrete specific vasoactive molecules, which may be related to the anticontractile characteristics of PVAT regions.

### 1.3 The adiponectin-eNOS pathway activity in regional PVAT adipocytes

eNOS also known as (NOS3 or NOSIII) is an enzyme named after the cell type (endothelial cell) in which it was first identified (Xia et al., 2016; Loesch and Dashwood, 2020). NO, a gasotransmitter and potent vasodilator primarily synthesized by endothelial cells, regulates vascular tone and maintains blood homeostasis (Brown et al., 2014; Nava and Llorens, 2016). The eNOS enzyme is widely recognized for its crucial role in the production of vasoprotective NO. Although this enzyme is expressed primarily in endothelial cells, it is not fully expressed in endothelial cells. In fact, recent studies have shown that eNOS is also expressed in PVAT, both of which help regulate vascular pathophysiology (Xia et al., 2016; Loesch and Dashwood, 2020). Under normal conditions, PVAT eNOS produces NO, which contributes to Ach-induced vasodilation. However, the role of PVAT eNOS may be more important than that of endothelial eNOS in obesity-induced vascular dysfunction (Xia et al., 2016; Xia and Li, 2017). A study conducted by Xia indicated that the endothelium-dependent, NO-mediated vasodilator response to Ach was unaltered in aortas from mice subjected to a high-fat diet. Notably, the presence of PVAT significantly attenuated the vasodilator response to Ach in the aorta of obese animals compared with that of lean controls (Xia et al., 2016). These findings further confirmed that eNOS in the PVAT plays a crucial role in vascular dysfunction of the aortas in mice fed a high-fat diet. However, a study has shown that inhibiting NOS does not block the anticontractile function of aortic PVAT. Consequently, researchers concluded that the relaxation factor derived from PVAT is not NO (Löhn et al., 2002). Experiments involving the transfer of organ bath solution have confirmed that stimulated PVAT releases a transferable anticontractile factor, namely, adiponectin (Saxton et al., 2018). Subsequently, several studies have also confirmed the anticontractile function of adiponectin in PVAT. The application of exogenous adiponectin to mouse mesenteric arteries constricted with NA mimics the anticontractile effect of PVAT (Lynch et al., 2013). The inhibition of adiponectin receptor-1 completely abolished the anticontractile effect of NA-induced PVAT (Greenstein et al., 2009; Lynch et al., 2013). Moreover, in adiponectin-deficient mice, the β3-adrenergic receptor agonist CL-316243 failed to elicit the PVAT-dependent hyperpolarization observed in control mice (Weston et al., 2013); undoubtedly, adiponectin, which is an adipokine secreted by PVAT, is a potent vasodilator (Lynch et al., 2013; Weston et al., 2013; Xia and Li, 2017).

Yudkin et al., (2005) coined the term “vasocrine” in 2005 to specifically refer to the paracrine communication between PVAT and its surrounding arteries. This term refers to certain adipokines that are released from adipocytes located in close anatomical proximity to the vessel (within the PVAT) and subsequently reach the tunica media, endothelium, and bloodstream. This phenomenon allows these molecules to exert a more direct and localized impact on the vascular wall, thereby influencing vascular function (Yudkin et al., 2005). AT-derived adiponectin exerts its effects directly on vascular smooth muscle cells (VSMCs), causing membrane hyperpolarization and inducing vasodilation (Badimon et al., 2017). Weston et al. (2013) also demonstrated that adiponectin stimulates the release of NO from adjacent adipocytes via a paracrine mechanism. This adipocyte-derived NO increases BKCa opening in VSMCs, which causes membrane hyperpolarization and vasodilation (Weston et al., 2013; Withers et al., 2014a; Withers et al., 2014b). Furthermore, the downstream signaling molecules associated with adiponectin play corresponding roles in the anticontractile effects induced by PVAT. AMP kinase (AMPK) is the primary downstream signaling target that is activated by adiponectin upon its interaction with AdipoR1 receptors (Yamauchi et al., 2007; Xu et al., 2010). AMPK stimulates the activity of eNOS by phosphorylating its Ser1177 site (Deng et al., 2009). Phosphorylation is a prominent posttranslational modification, with Ser1177 phosphorylation being the principal positive modulator of eNOS activity (Heiss and Dirsch, 2014). Consequently, adiponectin induces vasorelaxation through a mechanism dependent on the activation of AMPK (Almabrouk et al., 2017). The activation of the AMPK/eNOS pathway in PVAT adipocytes is responsible for their anticontractile function (Sun et al., 2014; Chen et al., 2016).

Previous studies conducted by Victorio indicated that the disparities in the anticontractile function of PVAT in the thoracic and abdominal regions were attributed to regional variations in PVAT eNOS-mediated NO production. The anticontractile effect of PVAT, however, is mediated through multiple pathways and mechanisms involving numerous vasoactive molecules. Furthermore, because PVATs in the thoracic and abdominal regions exhibit distinct phenotypes and have unique secretion characteristics (Emont et al., 2022), identifying the specific adipokines produced by PVAT and the signaling pathways (adiponectin-eNOS) involved in their contribution to regional PVAT is another basis for further evaluating the heterogeneity of the anticontractile function induced by PVAT in these regions.

### 1.4 Norepinephrine content in regional PVAT

**FIGURE 1:**
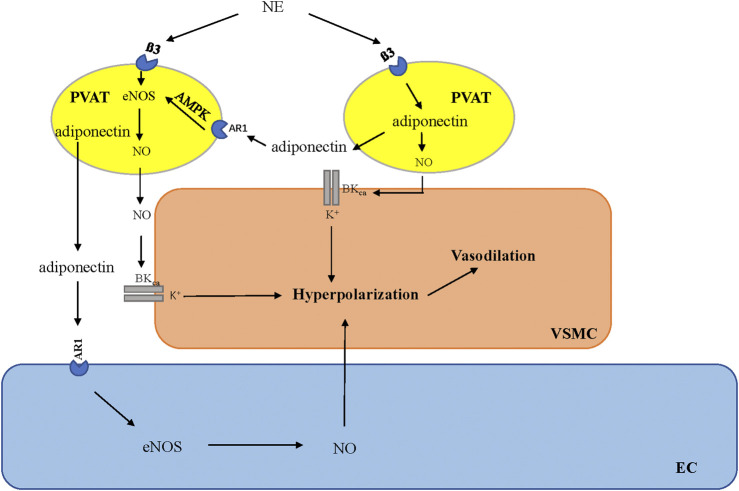
β3-Adrenoceptor is activated by norepinephrine (NE) derived from sympathetic nerves, which leads to the stimulation of adiponectin secretion from adipocytes in PVAT and the regulation of vascular tone through downstream signaling pathways. AMPK, adenosine monophosphate-activated protein kinase; eNOS, endothelial nitric oxide synthesis; NO, nitric oxide; PVAT, perivascular adipose tissue; VSMC, vascular smooth muscle cells; EC, endothelial cells.

PVAT contains an independent adrenergic system that can take up, metabolize, release, and potentially synthesize the vasoactive catecholamine norepinephrine (NE) (Ahmad et al., 2019). Previously, metabolic function was thought to be the only role for adrenoceptors on adipocytes (Dessy and Balligand, 2010); however, recent evidence suggests that the function of the β3-adrenoreceptor on adipocytes varies depending on the type of adipose tissue. In WAT, catecholamines induce lipolysis through β3-adrenoreceptors on adipocytes (Bartness et al., 2014; Robidoux et al., 2006). In BAT, β3-adrenoceptors stimulate thermogenesis (Cypess et al., 2015). The latest evidence suggests that the activation of β3-adrenoreceptors in the PVAT is involved in the regulation of vascular function (Sheng et al., 2016). Using β3-adrenoreceptor antagonists, it was confirmed that β3-adrenoreceptors play a crucial role in the anticontractile function of PVAT, and stimulation of β3-adrenoreceptors can induce PVAT to release the vasodilator adiponectin (Saxton et al., 2018). The mechanism by which PVAT exerts anticontractile function via beta3-adrenergic receptors has been confirmed in the mesenteric PVAT of mice (Table 1). Under basic conditions, adiponectin, a hyperpolarizing factor, is released in the mesenteric artery of intact PVAT mice under stimulation by the β3-adrenergic receptor, which can hyperpolarize underlying VSMCs by activating the BKCa 1.1 channel, releasing NO from adipocytes and inducing vasodilation (Weston et al., 2013) (Figure 1). This mechanism is driven by the sympathetic neurotransmitter NE (Hanscom et al., 2024). The response of adipocytes to NE and the biologically active role of PVAT in regulating vascular tone suggest that PVAT is functionally innervated (Soltis and Cassis, 1991; Agabiti-Rosei et al., 2014; Schleifenbaum et al., 2010; Zaborska et al., 2016; Saxton et al., 2018; Abu Bakar et al., 2017; Perdikari et al., 2022; Willows et al., 2021).

**TABLE 1:** Norepinephrine (NE) stimulates PVAT to release vasoactive factors to regulate vascular function.

Location of PVAT	Stimulus	PVAT-derived factors	Effects	Species	Condition	References
MA	NE	NO	Anti-contraction	Rat	Physiological	Bussey et al. (2018)
MA	NE	Adiponectin	Anti-contraction	Mouse	Obesity	Agabiti-Rosei et al. (2014)
TA; MA	NE	NO	Anti-contraction	Rat	Sepsis	Barp et al. (2020)
MA	NE	NO	Anti-contraction	Mouse	Obesity	Gil-Ortega et al. (2014)
MA	NE	Adiponectin	Anti-contraction	Mouse	Physiological	Lynch et al. (2013)
MA	NE	Adiponectin	Anti-contraction	Mouse	Hypoxia	Rosei et al. (2015)
MA	NA	Adiponectin	Anti-contraction	Rat	Physiological	Saxton et al. (2019b)
MA	NE	Adiponectin	Anti-contraction	Mouse	Physiological	Withers et al. (2017)
MA	EFS and EX	Adiponectin	Anti-contraction	Mouse	Obesity and Physiological	Saxton et al. (2021)
MA	EFS	Adiponectin	Anti-contraction	Mouse	Physiological	Saxton et al. (2018)
MA	NE	NO	Anti-contraction	Mouse	Hypertension	Persson, Marchetti, and Friederich-Persson, (2023)
MA	NE	NO	Anti-contraction	Rat	Obesity	Zaborska et al. (2016)

MA, mesenteric artery; TA, thoracic aorta; NO, nitric oxide; NE, norepinephrine; NA, noradrenaline; ADRB3, beta-3, adrenergic receptor; EFS, electrical field stimulation; EX, exercise.

The initial discovery of NE, a sympathetic neurotransmitter, in adipose tissue was in WAT (Vargovic et al., 2011; Kvetnansky et al., 2012); subsequently, the presence of catecholamine in the PVAT was also reported by Ayala-Lopez. Interestingly, the content of NE in adipose tissue exhibits regional differences. Ahmad et al. (2019) analyzed the NE content in PVAT (including thoracic PVAT, superior mesenteric PVAT, mesenteric PVAT, and retroperitoneal fat) and reported that thoracic PVAT has a high NE content, whereas mesenteric PVAT (mPVAT) has a low NE content, with the NE content in superior mesenteric PVAT being between that in thoracic PVAT and mesenteric that in PVAT. The NE content in thoracic PVAT is 7 times greater than that in mPVAT. The differences in NE content among different tissues may be related to the phenotype of the adipose tissue (Ahmad et al., 2019). Jeong et al, (2018) confirmed that the reason for the high level of NE in thoracic PVAT is that BAT is considered a heat-producing tissue driven by the sympathetic nervous system, resulting in greater NE content. While thoracic PVAT, contains more brown adipocytes, abdominal PVAT contains a mixture of cells, with unilocular white adipocytes being the main component and brown adipocytes making up a small portion (Police et al., 2009). The NE content in abdominal PVAT may be lower than that observed in thoracic PVAT. Further investigations are warranted to validate this hypothesis.

Additionally, because NE, a sympathetic transmitter, can stimulate the anticontractile effect of PVAT, substantial evidence suggests that PVAT is innervated by sympathetic nerves (Soltis and Cassis, 1991; Schleifenbaum et al., 2010; Agabiti-Rosei et al., 2014; Zaborska et al., 2016; Abu Bakar et al., 2017; Saxton et al., 2018; Willows et al., 2021; Perdikari et al., 2022). Sympathetic innervation is necessary not only for lipolysis but also for maintaining the thermogenic capacity of brown adipocytes, and nerve density may affect this capacity (Ayala-Lopez and Watts, 2017). Similarly, the density of nerves in adipose tissue also varies by region. Contreras et al. (2014) demonstrated that nerve density is crucial for the development of the brown fat cell phenotype (Contreras et al., 2014). In the absence of sustained sympathetic nerve stimulation, brown adipocytes in the inguinal adipose tissue of young mice transform into white adipocytes (Contreras et al., 2014). Additionally, an analysis of the density of innervation in aortic PVAT, mPVAT, and WAT revealed that sympathetic nerves densely innervated exclusively aortic PVAT consisting of brown adipocytes, whereas they did not densely innervate mPVAT composed of white adipocytes or WAT (Hanscom et al., 2024). Therefore, BAT contains more sympathetic nerve fibers than WAT does (Diculescu and Stoica, 1970; Sheng et al., 2016). Sympathetic inputs to WAT are less active than those to BAT (Ayala-Lopez and Watts, 2017). Thus, the innervation density of brown-like thoracic PVAT depots may be greater than that of abdominal PVAT depots containing a mixture of brown and white tissue, which may be related to the greater concentration of NE in thoracic PVAT. There are differences in sympathetic innervation and NE concentrations in PVAT in different regions, whereas the release of anticontractile factors by PVAT is driven by downstream processes in which NE stimulates adipocytes (Hanscom et al., 2024). The difference in NE content between thoracic and abdominal PVAT may contribute to the differences in the anticontractile function of PVAT in different regions by influencing disparities in the activity of the β3-adiponectin-NO pathway.

### 1.5 Discussion

The innervation of adipose tissue is a concept that was developed in the early 1960 s, and involves the potential adipose lipomotor neurons in the sympathetic nerve chain (Bartness and Bamshad, 1998; Correll, 1963). This long-standing concept, combined with the observation that NE (a sympathetic neurotransmitter) stimulates anticontractile effects of PVAT, led to the hypothesis that PVAT is innervated by sympathetic neurons (Hanscom et al., 2024). Since then, several studies have tested this concept. The removal of PVAT from the aorta or mesenteric artery enhances the contractile response to catecholamines, leading to increased vasoconstriction (Soltis and Cassis, 1991; Saxton et al., 2018). Pharmacological denervation mitigates the EFS-induced anticontractile effects of PVAT (Saxton et al., 2018; Soltis and Cassis, 1991; Mandel et al., 2013; Saxton et al., 2022). These studies have shown that the sympathetic-driven anticontractile effect of PVAT disappears after sympathetic denervation (Saxton et al., 2018; Sullivan and Davison, 2001; Saxton et al., 2019a; Török et al., 2016). This loss of sympathetic input coincides with the impairment of PVAT anticontractile function, suggesting that sympathetic innervation serves as the connection between electrical stimulation and the anticontractile function of adipocytes (Hanscom et al., 2024). However, the connection between nerves and adipocytes has long been controversial. While several studies have indicated a high degree of direct contact between nerves and adipocytes (Wirsen, 1964; Lever et al., 1986; Rebuffé-Scrive, 1991), others believe that only 2%–3% of adipocytes are directly innervated (Slavin and Ballard, 1978). To establish conclusive evidence for direct, classical communication between nerves and perivascular adipocytes mediated by NE, Hanscom et al. (2024). directly verified the hypothesis that adipocytes in PVAT are innervated by using contemporary anatomical markers and imaging techniques as well as calcium imaging studies of eurotransmission in PVAT. The use of contemporary anatomical methods to verify the results revealed that the nerve fibers traversing PVAT are related mainly to the vasculature and that there is no obvious anatomical connection with adipocytes (Hanscom et al., 2024). This finding suggests that while nerve fibers are present, they are not fully distributed in the adipose itself, suggesting limited innervation (Hanscom et al., 2024). Functional experiments have shown that although adipocytes are highly reactive to NE, application of the maximum stimulus to nerves in PVAT causes a minimal response from adipocytes. This finding suggests that PVAT is not innervated in the same ways as other tissues (Hanscom et al., 2024). In addition, the results of studies examining the functional neuroregulation of adipocyte responses by measuring the adipocyte calcium response following EFS-induced nerve depolarization suggest that *in vitro* EFS of abdominal PVAT and mPVAT does not induce a strong calcium response in adipocytes themselves (Hanscom et al., 2024). This limited neuroregulation of adipocytes indicates a lack of functional connectivity between neurons and adipocytes. A comprehensive analysis revealed that PVAT is not innervated in the typical manner. These findings challenge the commonly held view that sympathetic innervation regulates adipocyte function and drives the anticontractile effect of PVAT and highlight other mechanisms that control adrenergic anticontractile function in PVAT (Hanscom et al., 2024). These observations suggest that the regulation of the anticontractile function of PVAT by the adrenergic system is independent of neural control and arises from nonneural mechanisms (Hanscom et al., 2024), highlighting the importance of exploring alternative mechanisms that may play a role in regulating adipocyte function.

Although the results of this study suggest that PVAT is not innervated and that NE is unlikely to be neurogenic, the catecholamine system present within adipocytes (Pizzinat et al., 1999). Pizzinat et al. (1999) first demonstrated the expression of monoamine oxidases A and B in adipocytes, which indicates that adipocytes can take up and metabolize monoamine substances such as NA. Research by Saxton et al. (2018) also confirmed that PVAT has a dual role. In addition to releasing the anticontractile factor adiponectin to regulate vascular function, it can also take up exogenous catecholamines, acting as a reservoir of NA, preventing NA from reaching blood vessels and causing contraction (Saxton et al., 2018). As a reservoir for exogenous NE, changes in the uptake and storage of NE by PVAT adipocytes may also have profound effects on vascular tone. Therefore, understanding the mechanism of PVAT NA metabolism and its effect on the ß3-adiponectin-NO-mediated anticontractile effect in adipocytes is another indicator for further verification of the differences in regional PVAT anticontractile function.

### 1.6 Conclusion

In conclusion, compared with that of the thoracic aorta, the anticontractile function of the abdominal PVAT is impaired. This is due to the regional phenotypic differences in PVAT in different vascular bed regions, which may lead to increased heterogeneity in NE content and secretion profiles, and subsequently affect the regulation of specific adipokine signaling pathways in regional PVAT, thereby leading to differences in vascular function. On the basis of the heterogeneity of regional PVAT, this review focuses on the potential role of vasoactive factors secreted by regional PVAT in the regulation of vascular tone in rodents. With respect to the existing relevant studies, most of the information regarding the signaling between PVAT and the underlying vascular wall comes from studies using rodent models. As in rodents, phenotypic variations in PVAT exist around the aorta in humans. The PVAT in the thoracic aorta exhibits the characteristics of BAT, which has thermogenic and anti-inflammatory effects (Shi et al., 2022), whereas the abdominal PVAT is a mixture of brown and white fat (Folkesson et al., 2017) that contains many inflammatory markers that which may explain the increased susceptibility to diseases in the abdominal aorta region (Meekel et al., 2021; Folkesson et al., 2017). Owing to the unique anatomical characteristics of PVAT adjacent to blood vessels, it plays a crucial role in the maintenance of normal cardiovascular function and the development of cardiovascular disease. Moreover, because of the highly invasive nature of aortic PVAT acquisition (Lehman et al., 2010), parallel studies in humans are challenging and lacking. In the current social environment, in which cardiovascular disease is mainstream on the spectrum of human illnesses the study of PVAT, which is closely related to cardiovascular health, is useful for understanding the mechanism of onset and development of cardiovascular disease and for undertaking a series of interventions to improve people’s quality of life.
